# The role of N-acetylcysteine on adhesion and biofilm formation of Candida parapsilosis isolated from catheter-related candidemia

**DOI:** 10.1099/jmm.0.001848

**Published:** 2024-07-03

**Authors:** Xiao-shu Zuo, Qian-yu Wang, Sha-sha Wang, Guang Li, Li-ying Zhan

**Affiliations:** 1 Department of Critical Care Medicine, Renmin Hospital of Wuhan University, Wuhan 430060, Hubei Province, PR China; 2 Department of Clinical Laboratory, Renmin Hospital of Wuhan University, Wuhan 430060, Hubei Province, PR China

**Keywords:** adhesion, biofilm formation, *Candida parapsilosis*, candidemia, *N*-acetylcysteine

## Abstract

**Objectives.** Anti-fungal agents are increasingly becoming less effective due to the development of resistance. In addition, it is difficult to treat *Candida* organisms that form biofilms due to a lack of ability of drugs to penetrate the biofilms. We are attempting to assess the effect of a new therapeutic agent, *N*-acetylcysteine (NAC), on adhesion and biofilm formation in *Candida parapsilosis* clinical strains. Meanwhile, to detect the transcription level changes of adhesion and biofilm formation-associated genes (*CpALS6, CpALS7, CpEFG1* and *CpBCR1*) when administrated with NAC in *C. parapsilosis* strains, furthermore, to explore the mechanism of drug interference on biofilms.

**Hypothesis/Gap statement.** N-acetylcysteine (NAC) exhibits certain inhibitory effects on adhesion and biofilm formation in C. parapsilosis clinical strains from CRBSIs through: (1) down-regulating the expression of the CpEFG1 gene, making it a highly potential candidate for the treatment of C. parapsilosis catheter-related bloodstream infections (CRBSIs), (2) regulating the metabolism and biofilm -forming factors of cell structure.

**Methods.** To determine whether non-antifungal agents can exhibit inhibitory effects on adhesion, amounts of total biofilm formation and metabolic activities of *C. parapsilosis* isolates from candidemia patients, NAC was added to the yeast suspensions at different concentrations, respectively. Reverse transcription was used to detect the transcriptional levels of adhesion-related genes (*CpALS6* and *CpALS7*) and biofilm formation-related factors (*CpEFG1* and *CpBCR1*) in the *BCR1* knockout strain, CP7 and CP5 clinical strains in the presence of NAC. To further explore the mechanism of NAC on the biofilms of *C. parapsilosis*, RNA sequencing was used to calculate gene expression, comparing the differences among samples. Gene Ontology (GO) enrichment analysis helps to illustrate the difference between two particular samples on functional levels.

**Results.** A high concentration of NAC reduces the total amount of biofilm formation in *C. parapsilosis*. Following co-incubation with NAC, the expression of *CpEFG1* in both CP7 and CP5 clinical strains decreased, while there were no significant changes in the transcriptional levels of *CpBCR1* compared with the untreated strain. GO enrichment analysis showed that the metabolism and biofilm-forming factors of cell structure were all regulated after NAC intervention.

**Conclusions.** The non-antifungal agent NAC exhibits certain inhibitory effects on clinical isolate biofilm formation by down-regulating the expression of the *CpEFG1* gene, making it a highly potential candidate for the treatment of *C. parapsilosis* catheter-related bloodstream infections.

## Data Availability

The datasets analysed during the current study are available at the following link: https://www.ncbi.nlm.nih.gov/sra/PRJNA1100759. Also, all the other data are provided in full in the Results section of this article.

## Introduction

Clinical bloodstream infections caused by non-*Candida albicans Candida* have increased in recent years, with *Candida parapsilosis* becoming a secondary causative pathogen for nosocomial candidemia [1,3]. Studies have reported that the majority of cases with a *C. parapsilosis* infection were catheter-related bloodstream infections (CRBSIs) [4,6]. The results of the National Invasive Fungal Drug Resistance Surveillance Network also showed that *C. parapsilosis* had the highest isolation rate in blood and catheter specimens collected from patients with invasive candidiasis [7,8]. These infections can lead to high mortality, morbidity and increased medical costs for patients. Therefore, studies on the characterization of *C. parapsilosis* would improve the understanding of its pathogenic mechanism as well as lead to the development of methods for clinical anti-infection treatment to lower mortality.

The pathogenicity of *C. parapsilosis* infection is attributable to various factors including adhesion and the formation of a biofilm. *Candida* biofilms are extracellular matrices (ECM) that contain a large number of *Candida* cells that irreversibly adhere to biological or non-biological surfaces. This process helps the micro-organism evade anti-bacterial drugs and the host’s immune system. Biofilms also continue to release pathogens into the blood, causing long-term, recurrent infections in the body that are difficult remove completely [9,10]. Biofilm-associated infection has become a difficult clinical problem, and there is an urgent need to investigate the underlying mechanisms and develop clinical intervention drugs.

After the formation of a *Candida* biofilm, the sensitivity of antifungal drugs such as azoles and echinocandins is reduced by 10- or even 1000-fold [11]. In addition, the effect of non-antibiotic drugs on the formation of antifungal biofilms is receiving increased attention from clinical researchers. In this regard, *N*-acetylcysteine (NAC) has expectorant, antioxidant, anti-inflammatory, and other effects [12], with studies reporting that it inhibits bacterial adhesion, reduces the production of extra-cellular glycoproteins, and has specific anti-biofilm effects [13]. Related cell experiments have shown that NAC treatment can disrupt or alter biofilm formation by affecting adhesion and the matrix structure of biofilms, destroying existing biofilms and inhibiting the spread of the biofilm [14]. Taken together, these findings indicate that the anti-biofilm agent NAC has specific inhibitory effects on biofilm formation and degradation [15].

Based on the above background, this study focused on the effects of drug intervention on the adhesion ability and biofilm formation ability of clinical isolates of *C. parapsilosis* isolated from central venous catheter (CVC)-related bloodstream infections. The non-antifungal agent NAC with potential anti-*Candida* biofilm effects was selected to evaluate its ability to reduce adhesion and biofilm formation. We also investigated the mechanisms of NAC’s protective effects in order to find potential drugs to treat adhesion and biofilm formation in *C. parapsilosis*-related infections.

## Methods

### Strains

1. **Strain selection criteria:** The clinical strains were isolated from blood or catheter samples of patients with CRBSIs. The diagnostic criteria for *Candida* CRBSIs were defined in our previous study [16]. All the clinical isolates were cultured in accordance with the standard microbiologic method. Approval for the protocol was obtained from the Local Ethics Committee at Renmin Hospital of Wuhan University (RHWU).
2. **Basic information on research strains**: According to the **strain selection criteria** (1), six strains of *C. parapsilosis* were isolated from blood or catheter samples of CRBSI patients in the intensive care unit, and the relevant clinical information was recorded. The *C. parapsilosis* clinical strains used in this study can be found in our previous study [16].
3. **Gene detection strains**: Using the results of the adhesion and biofilm experiments, a CP7 strain with strong adhesion and biofilm formation ability and a CP5 strain with strong adhesion and biofilm formation ability were selected for the study of gene detection.
4. **Transcriptome sequencing strains**: A CP7 strain with strong adhesion and biofilm formation ability was selected for transcriptome sequencing before and after drug intervention.
5. **Control strains**: *Parapsilosis* standard strain ATCC 22019 and *BCR1* gene knockout strain (CpΔ*BCR1*) were used as controls. CpΔ*BCR1* was provided by Professor Buttler, University College Dublin, and showed less ability to form a biofilm formation after knockout of the *BCR1* gene [17].

### Antifungal susceptibility (MIC)

#### Strain identification and preparation

1. A fully automated microbial matrix-assisted laser desorption/ionization time-of-flight mass spectrometry (MALDI-TOF-MS) detection system was used for strain identification to ensure that all strains used in the experiment were pure.
2. The stored strains were transferred to liquid culture medium Sabouraud dextrose broth (SDB) for resuscitation, placed on a constant temperature shaker at 37 ℃ and 180 r.p.m. for 24 h.
3. The recovered strains were transferred to Sabouraud dextrose agar solid culture medium for 24 h of recovery.

#### MIC experiments

The MIC of NAC in six clinical isolates and one standard strain of *C. parapsilosis* was measured. Due to a lack of drug sensitivity breakpoints for non-antibiotic agents such as NAC, we set the drug concentration gradients based on published studies [14,15].

1. Drug stock solution and preparation: A stock solution of NAC (100 mg ml^−1^) was prepared in PBS followed by sterilization by 0.22 µM sterile filter membrane filtration. The stock solution was packaged in small quantities and stored at −80 ℃.
2. Experimental solution: NAC in Sensititre broth was diluted to obtain solutions with a concentration gradient of 25 mg ml^−1^, 10 mg ml^−1^, 5 mg ml^−1^, 2.5 mg ml^−1^, 1 mg ml^−1^, 0.5 mg ml^−1^, 0.25 mg ml^−1^ and 0.125 mg ml^−1^.
3. The micro broth dilution method was used for drug sensitivity testing: Several fully isolated colonies (diameter >1 mm) were selected from a 24 h pure culture of *C. parapsilosis* strains and emulsified in sterile water. The suspension was adjusted to a bacterial solution with a concentration of 0.5 McFarland using an electronic turbidimeter. A 20-µl aliquot of the suspension was then added to 11 ml broth to obtain an inoculum concentration of (1.5–8) ×10^3^ c.f.u. ml^−1^.
4. The diluted inoculum (100 µL per well) was to a 96-well drug-sensitive plate.
5. The *Candida* suspension and drug solution were co-incubated for 24 h and then the results were observed. The experiment was repeated at least three times.
6. MIC endpoint determination: The drug concentration with 50% inhibition of yeast growth (significantly reduced turbidity observed by the naked eye), compared with the control was classified as the MIC.

### Determination of NAC treatment concentration

1. Based on the results of relevant literature [14,15], we selected the concentration of NAC in the *C. parapsilosis* clinical isolates used to detect the effects on adhesion and biofilm formation.

### NAC intervention on adhesion ability

Referring to a protocol for evaluating the adhesion ability of yeast cells introduced by Silva *et al*. [18], flow cytometry was utilized to assess the impact of NAC intervention on the adhesion of *C. parapsilosis* isolates. The details are described in our previous article [16].

1. **Strain recovery**. The strains were resuscitated, incubated and then identified by MALDI-TOF-MS as the strain identification and preparation section.
2. **Drug intervention**. The strains were divided into three groups: drug-free group, low NAC concentration group and high NAC concentration group, and then incubated at 37 ℃ and 180 r.p.m., for 24 h.
3. **Flow cytometry was used to assess adhesion ability**. The packaged strains of the three groups were centrifuged at 3000 r.p.m. for 3 min. PBS (300 µL) and fluorescent microspheres (300 µL, 2×10^8^ microspheres/ml) were added to each tube separately and centrifuged at room temperature (25 ℃) at 150 r.p.m. for 30 min. The flow cytometry scanned 50 000 yeast cells for analysis.
4. **Analyze the results**. According to references [16,18], using the percentage of yeast cells bound to fluorescent microspheres (P2) in flow cytometry is a parameter that reflects adhesion. We classified the adhesion ability of different types of *Candida* clinical isolates into four levels: weak (P2 ≤20), moderate (20 < P2 ≤ 30), strong (30 < P2 ≤ 50) and very strong (P2 >50). The adhesion distribution modes were also divided into homogenic and heterogenic. A homogenous distribution pattern indicates yeast cells are bound to an equal amount of microspheres (frequently binding to a single microsphere). In contrast, a heterogeneous pattern displays the presence of multiple peaks and means adhering to more than one microsphere to each cell [16,18].

### NAC intervention on biofilm formation

The inhibition effect of NAC on *C. parapsilosis* biofilm was assessed using the XTT reduction assay and crystal violet assay, respectively. XTT (2,3-bis(2-methoxy-4-nitro-5-sulfophenyl)-5-toluidine-2H-tetrazolium) is a reduction agent. The tetrazolium salt (XTT) reduction assay is the test most commonly used to estimate viable biofilm growth and to examine the impact of biofilm therapies. XTT was performed as a reduction agent in 96-well microtiter plates to study the formation of Candida biofilm. CV (Crystal violet staining) is one of the most classic biofilm detection method.

1. Strain recovery and NAC intervention. Refer to Strain identification and preparation section.
2. The yeast suspension was mixed, and then 100 µL was added to a 96-well cell culture plate, followed by the addition of 100 µL of SDB medium to the control well and incubation at 37 ℃ for 12 h.
3. A 100-µL aliquot of SDB medium, low concentration NAC and high concentration NAC was added to SDB medium for each group and co-incubated at 37 ℃ for 24 h.
4. XTT staining: (1) The upper layer of the culture medium and yeast suspension were gently aspirated, washed 3 times with 200 µL sterile PBS, and the yeast cells that did not fully adhere to the bottom of the well were removed. (2) A 100-µL aliquot of the XTT reduction solution was added to each well and reacted for 2 h at 37 ℃ in a dark place. (3) The culture plate was removed, and the absorbance value (OD) read a wavelength of 450 nm on the enzyme reader, which positively reflected the metabolic activity of the biofilm cells. The above experiment was repeated at least three times.
5. CV staining: (1) The upper layer of the culture medium was gently removed, each well was rinsed with 200 µL of sterile PBS, and the procedure was repeated twice. (2) The cell plate was placed in a 37 ℃ oven for drying (approximately 1 h). (3) A 100-µL aliquot of 10% formaldehyde was added to each well and fixed at room temperature for 2 min, then the fixing solution was removed and left to dry. (4) 20 mg ml^−1^ of crystal violet dye solution (100 µL) was added to each well and incubated at 37 ℃ for 30 min. (5) The crystal violet dye solution was absorbed, washed with distilled water 3–5 times and the washing solution was removed. (6) A 100-µL aliquot of 95% ethanol was added to each well for decolorization, and the absorbance at OD 630 nm was measured. A positive correlation reflected the number of cells, which represented the total amount of biofilm.

### Detection of adhesion- and biofilm formation-related genes after drug intervention

Considering the increasing resistance of *C. parapsilosis* to Fluconazole (FZ) in clinical practice [19] and our previous findings that high concentrations of NAC (50 mg ml^−1^) may have an inhibition effect on adhesion and biofilm, we used quantitative reverse transcription (qRT-PCR) to detect the expression of adhesion-related genes (*CpALS6* and *CpALS7*) and biofilm formation-related genes (*CpEFG1* and *CpBCR1*) in *C. parapsilosis* after NAC intervention.

1. Strains. We used NAC-treated *BCR1*-deleted strains, CP5 and CP7 for the detection of gene transcription.
2. Biofilm collection. The strains were divided into a drug-free group and a high-concentration NAC group (50 mg ml^−1^) and then cultivated at 37℃ for 24 h. The upper culture medium was sucked out, followed by gently washing with PBS and the attached biofilm was scraped out and transferred to a 1.5- ml Eppendorf tube (EP) for centrifugation at 7500 r.p.m. for 15 min.
3. Total-RNA extraction. Total RNA was extracted from the yeast cells using TRIzol Reagent according to the manufacturer’s instructions (Invitrogen) and then genomic DNA was removed using DNase I (TaKara). The RNA quality was determined using a 2100 Bioanalyser (Agilent) and quantified using the ND-2000 (NanoDrop Technologies). Only high-quality RNA samples (OD 260/280 = 1.8~2.0, OD 260/230 ≥2.0, RNA integrity number ≥6.5, 28S:18S ≥1.0, ≥100 ng/µL, ≥2 µg) were used to construct the sequencing library.
4. cDNA synthesis. The reverse transcription kit was used to reverse the extracted total RNA into cDNA, according to the instructions of the manufacturer.
5. The real-time PCR procedure was used to detect the expression of biofilm- and adhesion-related genes (*CpALS7*, *CpALS6*, *CpEFG1* and *CpBCR1*) after NAC administration. The qRT-PCR primer sequences for the adhesion- and biofilm formation-related genes are listed in Table 1.

**Table 1.** qRT-PCR primer sequences of adhesion and biofilm formation related genes

Primers	Up sequences (5’~3’) and down sequences (5’~3’)
*CpACT1*	AGTGTGACTTGGATGTCAGAAAGGAATTGT ACAGAGTATTTTCTTTCTGGTGGAGCA
*CpEFG1*	GTTCATACTATCAAGGTATGGAGGAGC GGTATTGGTATGGTAAGACGA
*CpBCR1*	GTCAGGGACATCACAAGTACTTC GGTGGCAATGGAGGTAAACTA
*CpALS7*	TCGAGTTCCTAATGGTGCAG CCTTCTTCACCCCAGTTTTG
*CpALS6*	CGGCGCAGATGTGCTAATG AAAGTCACCACCACCGAGG

*CpACT1*:： reference gene.

### RNA sequencing

1. **RNA extraction.** Refer to Detection of adhesion- and biofilm formation-related genes after drug intervention section
2. **Library construction and sequencing**. The RNA-seq transcriptome library was prepared using the TruSeq™ RNA sample preparation kit from Illumina (San Diego, CA, USA) using 2 µg of total RNA. Ribosomal RNA depletion instead of poly(A) purification was performed by the Ribo-Zero Magnetic kit (Epicentre) and then all the mRNAs were broken into short (200 nt) fragments by adding fragmentation buffer. Secondly, double-stranded cDNA was synthesized using a SuperScript double-stranded cDNA synthesis kit (Invitrogen) with random hexamer primers (Illumina). When the second strand of cDNA was synthesized, dUTP (2'-deoxyuridine 5'-triphosphate) was incorporated in place of dTTP (2'-deoxythymidine 5'-triphosphate). The synthesized cDNA was then subjected to end-repair, phosphorylation and ‘A’ base addition according to the Illumina library construction protocol. The second strand cDNA with dUTP was identified and degraded by the uracil-DNA glycosylase enzyme. The libraries were size selected for cDNA target fragments of 200 bp on 2% low-range ultra agarose gels followed by PCR amplification using Phusion DNA polymerase (NEB) for 15 PCR cycles. After quantification by the TBS-380 Mini-Fluorometer, the paired-end RNA-seq sequencing library was sequenced with the Illumina HiSeq×TEN (2×150 bp read length). The processing of the original images into sequences, base-calling and quality value calculations was performed using the Illumina GA Pipeline (version 1.6), in which 150 bp paired-end reads were obtained. A Perl programme was written to select clean reads by removing low-quality sequences, reads with more than 5 % of N bases (unknown bases) and reads containing adaptor sequences.
3. **Bioinformatics analysis**. The data generated by the Illumina platform were used for the bioinformatics analysis. All of the analyses were performed using the free online platform of Majorbio Cloud Platform (www.majorbio.com), produced by Shanghai Majorbio Bio-Pharm Technology Co., Ltd. The raw sequencing reads were deposited into the National Center for Biotechnology Information Sequence Read Archive (SRA) database (accession number: SRP501976). SRA records will be accessible with the following link: https://www.ncbi.nlm.nih.gov/sra/PRJNA1100759.
4. **Mapping reads to the reference genome**. High-quality reads in each sample were mapped to the reference genome provided by the customer. The analysis tool used was Bowtie2 (http://bowtie-bio.sourceforge.net/bowtie2/index.shtml).

### Statistical analysis

GraphPad Prism 6 software was used for the statistical analysis, with the results expressed as mean ± standard error (mean ± SEM). A students t-test was used for comparison between two groups, a one-way ANOVA was used for comparison between multiple groups and the Holm–Sidak test was used for multiple comparison tests based on analysis of variance. *P* < 0.05 indicated a statistically significant difference.

## Results

### Microbiological susceptibility of *C. parapsilosis*

As shown in Table 2, the MIC concentration of NAC for *C. parapsilosis* clinical isolates was 2.5–10 mg ml^−1^.

**Table 2.** MIC of NAC on *C. parapsilosis* isolates

Strains	MIC of NAC (mg/ml)
Standard	5.0
*parapsilosis* 5	2.5
*C. parapsilosis* 6	2.5
*C. parapsilosis* 7	2.5
*C. parapsilosis* 8	10.0
*C. parapsilosis* 9	5.0
*C. parapsilosis* 10	5.0

### NAC effects on adhesion of *C. parapsilosis*

Four adhesion profiles were established to reduce the variability in adherence to microspheres (P2) in different *Candida* isolates and better evaluate the adhesion strength of each isolate. Based on the flow cytometry results the profiles were classified as weak (P2: 1–20), medium (P2: 21–30), strong (P2: 31–50) and very strong (P2: >50). The adhesion characteristics of the six *C. parapsilosis* isolates are listed in Table 3.

**Table 3.** Adhesion characteristics of *C. parapsilosis* isolates

Strain type	Adhesion		
	P2	Distribution mode	Adhesion patterns
Standard	48.3	Heterogenic	Strong
*C. parapsilosis* 5	40.6	Heterogenic	Strong
*C. parapsilosis* 6	77.6	Heterogenic	Very strong
*C. parapsilosis* 7	89.1	Heterogenic	Very strong
*C. parapsilosis* 8	51.8	Heterogenic	Very strong
*C. parapsilosis* 9	50.5	Heterogenic	Very strong
*C. parapsilosis* 10	56.4	Heterogenic	Very strong

**Fig. 1.**
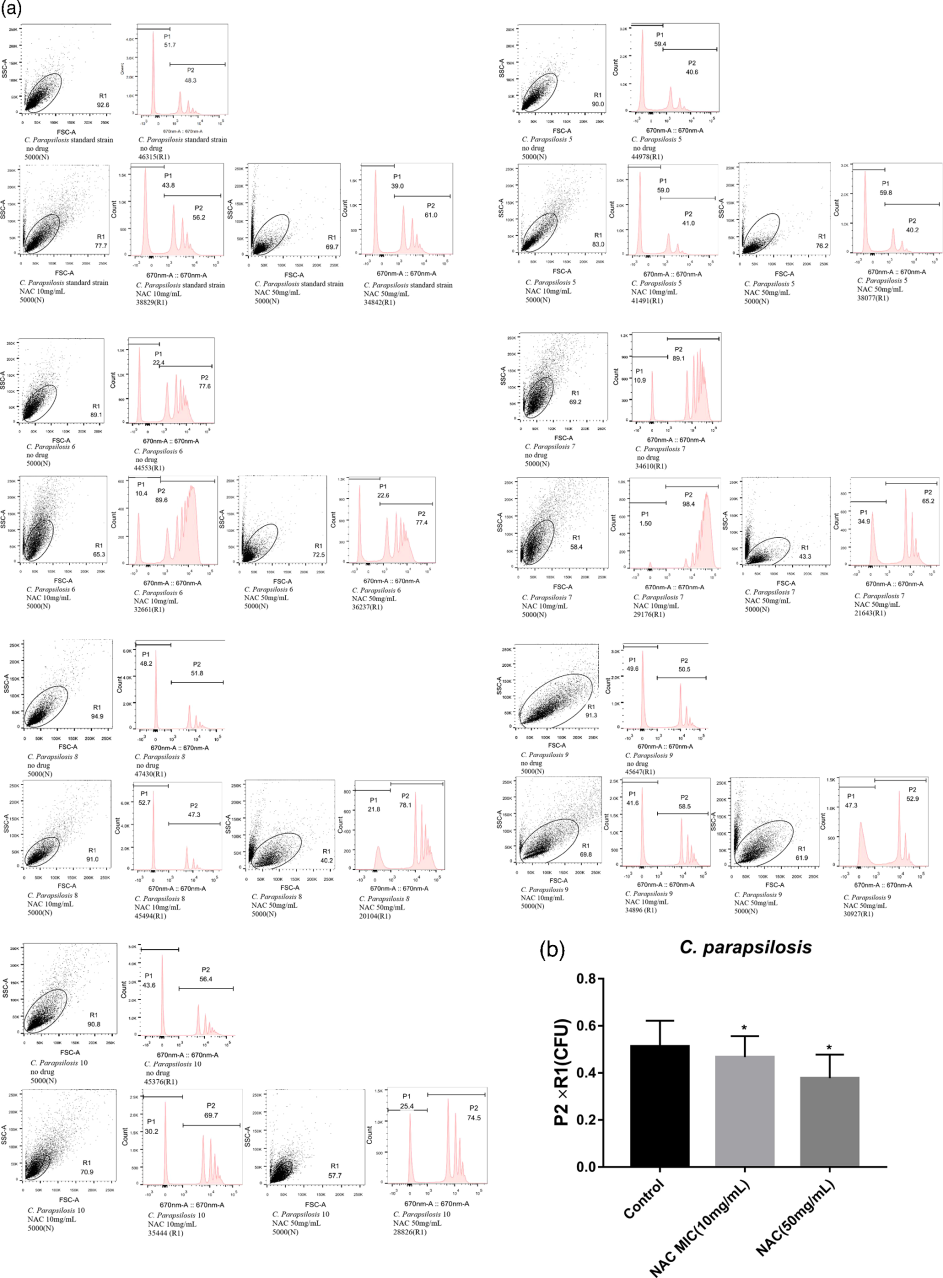
(a) Flow cytometry patterns of *C. parapsilosis* before and after the action of NAC at different concentrations. P2: Percentage of yeast cells bound to microspheres. P1: Percentage of yeast cells not bound to microspheres. The isolates detected in the figure are *C. parapsilosis* 5 (heterogenic, strong), 6 (heterogenic, very strong), 7 (heterogenic, very strong), 8 (heterogenic, very strong), 9 (heterogenic, very strong) and 10 (heterogenic, very strong). (b) Quantification of the anti-biofilm effect in (a). ‘P2×R1’ in (a) is used to represent c.f.u. *P < 0.05; **P < 0.01; ***P < 0.001; ****P < 0.0001.

Based on these adhesion patterns, almost all *C. parapsilosis* isolates displayed a very strong profile, with only one strain (Cpa 5) showing strong adhesion (Fig. 1a).

Using the flow cytometry results after drug action, a c.f.u. count (P2×R1) was selected to evaluate the inhibitory effect of NAC on the adhesion of *C. parapsilosis*. Both concentrations of NAC inhibited the adhesion of *C. parapsilosis* to a certain extent (Fig. 1b).

**Fig. 2.**
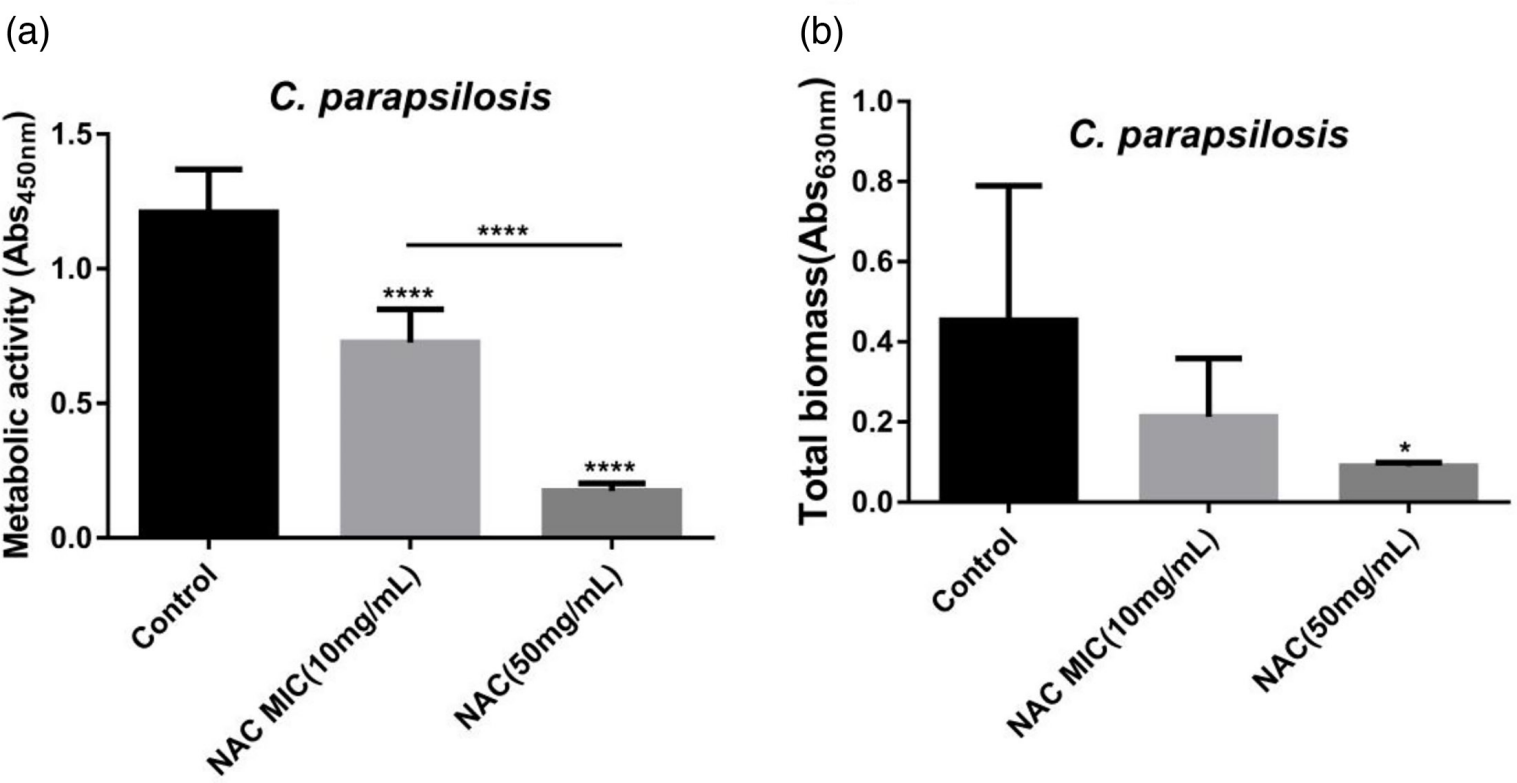
(a, b) XTT and CV results of *C. parapsilosis* against NAC at both MIC and high concentrations. The XTT reduction method is used for measuring the metabolic activity of biofilms. The crystal violet method is used to detect the total amount of biofilm. Six clinical strains of *C. parapsilosis* used in this experiment were isolated from CRBSI patients in the intensive care unit. Each strain was tested three times independently. *P < 0.05; **P < 0.01; ***P < 0.001; ****P < 0.0001.

To compare the total biofilm formation of *C. parapsilosis* isolates, we performed a CV assay to measure the total biomass of the biofilms at 24 h. The results indicated that only a high concentration of NAC reduced the total amount of biofilm formation by *C. parapsilosis* (Fig. 2b). The XTT [sodium salt; 2,3-bis-(2-methoxy-4-nitro-5-sulfophenyl)-2H-tetrazolium-5-carboxanilide] results demonstrated that both concentrations of NAC had an inhibitory effect on adhesion.

The biofilm metabolic activity of the *C. parapsilosis* strain decreased significantly after exposure to NAC (Fig. 2a), with the activity of the group exposed to a high concentration of NAC being stronger than that of the low concentration group (Fig. 2a).

### NAC effects on the expression of adhesion- and biofilm formation-related genes

qRT-PCR was used to detect the transcriptional levels of the adhesion-related genes (*CpALS6* and *CpALS7*) and biofilm formation-related genes (*CpEFG1* and *CpBCR1*) in the *BCR1* knockout strain (*Cp*∆*BCR1*) and the CP7 and CP5 clinical strains following incubation with NAC.

**Fig. 3.**
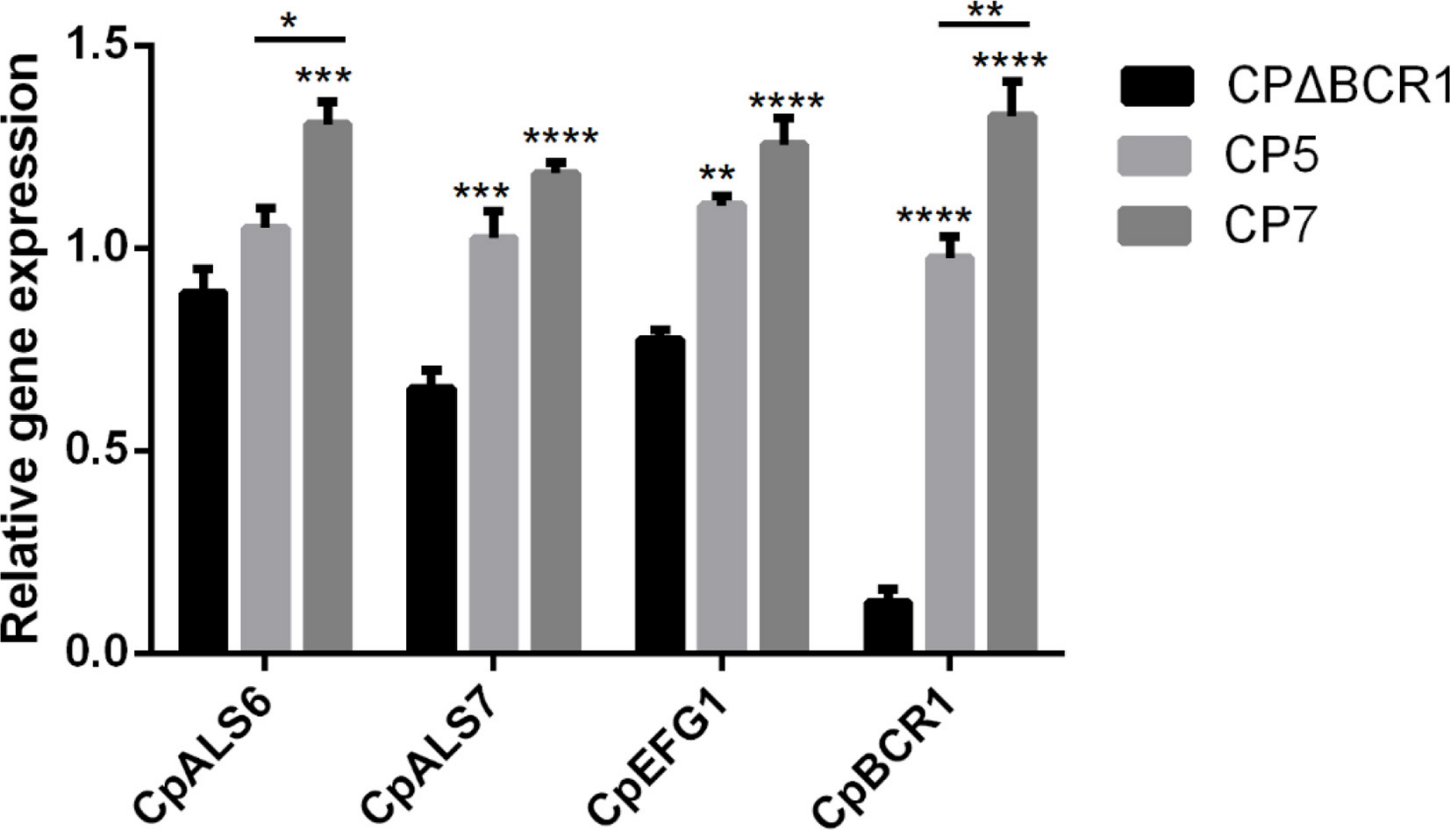
Relative expression of genes related to adhesion and biofilm formation in different *C. parapsilosis* isolates. Using CpACT1 as the reference gene, the transcriptional level of the target gene was determined by qRT-PCR. Compared with BCR1 experimental knockout strains with weak biofilm formation ability, *C. parapsilosis* clinical strains CP5 and CP7 with different adhesion and biofilm abilities were determined. *P < 0.05; **P < 0.01; ***P < 0.001; ****P < 0.0001.

The qRT-PCR results showed that the expression of two genes encoding the adhesins *CpALS6* and *CpALS7* and two biofilm-related genes *CpEFG1* and *CpBCR1* was significantly higher in the CP7 clinical strain compared with that in the *BCR1* gene knockout mutant strain. Moreover, the expression of *CpALS7*, *CpEFG1* and *CpBCR1* in CP5 was higher than that in the mutant strain. Of all the genes, the expression of *CpALS6* and *CpBCR1* in CP7 was higher than that observed in CP5 (Fig. 3).

**Fig. 4.**
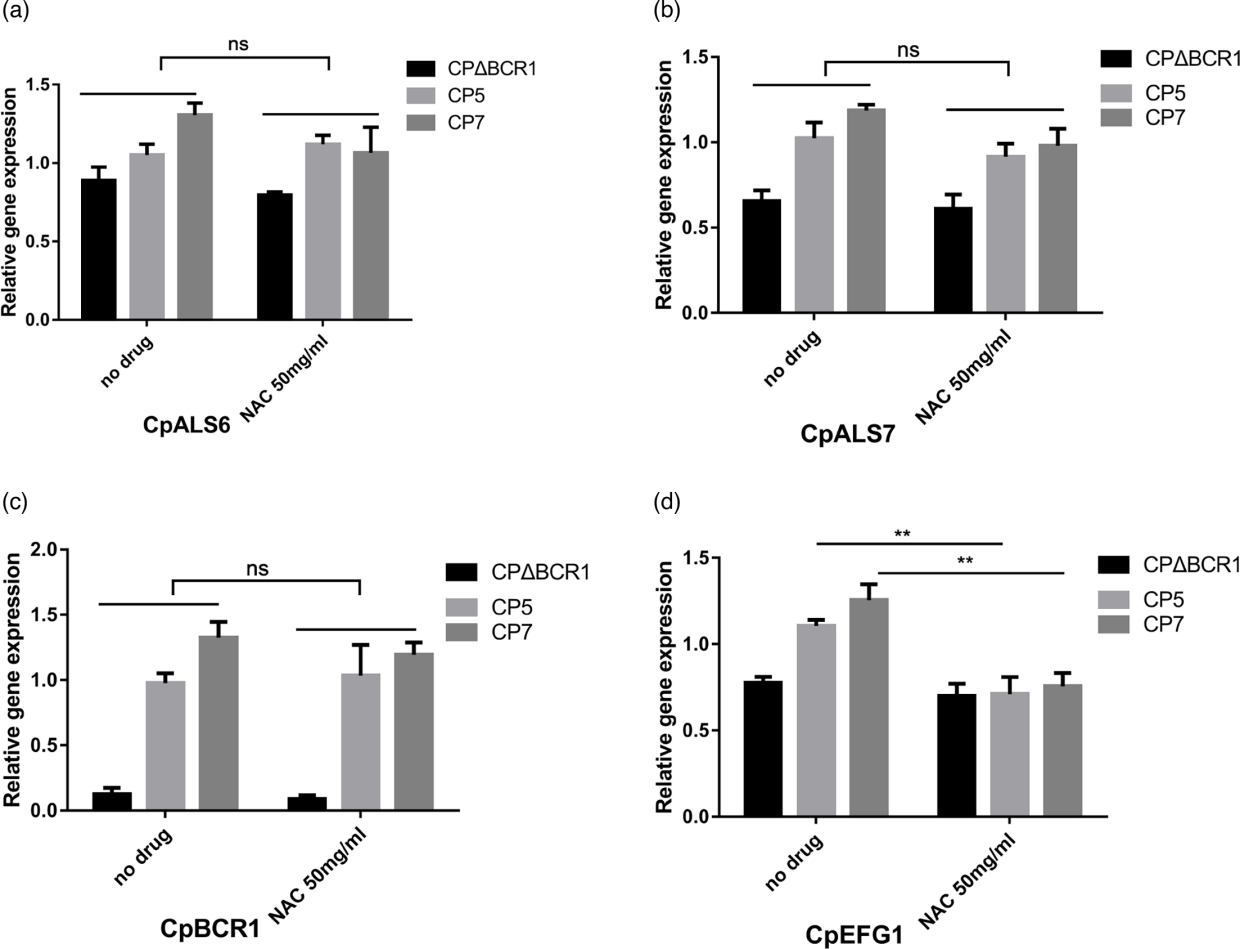
Relative expression of biofilm-related genes in *C. parapsilosis* treated with NAC. The clinical strains CP5 and CP7 with strong and very strong biofilm forming ability were determined, and the BCR1 gene knockout strain (Cp∆BCR1) with weak biofilm forming ability was selected as control. CpACT1 was used as a reference gene, and the transcriptional level of target gene was determined by qRT-PCR. *P < 0.05; **P < 0.01; ***P < 0.001; ****P < 0.0001.

Following co-incubation with NAC, the expression of *CpEFG1* in both the CP7 and CP5 clinical strains was decreased, while no significant changes in transcriptional levels of *CpBCR1* were observed compared with that measured in the untreated strain (Fig. 4).

### Effects of NAC on the transcriptome of *C. parapsilosis*

**Fig. 5.**
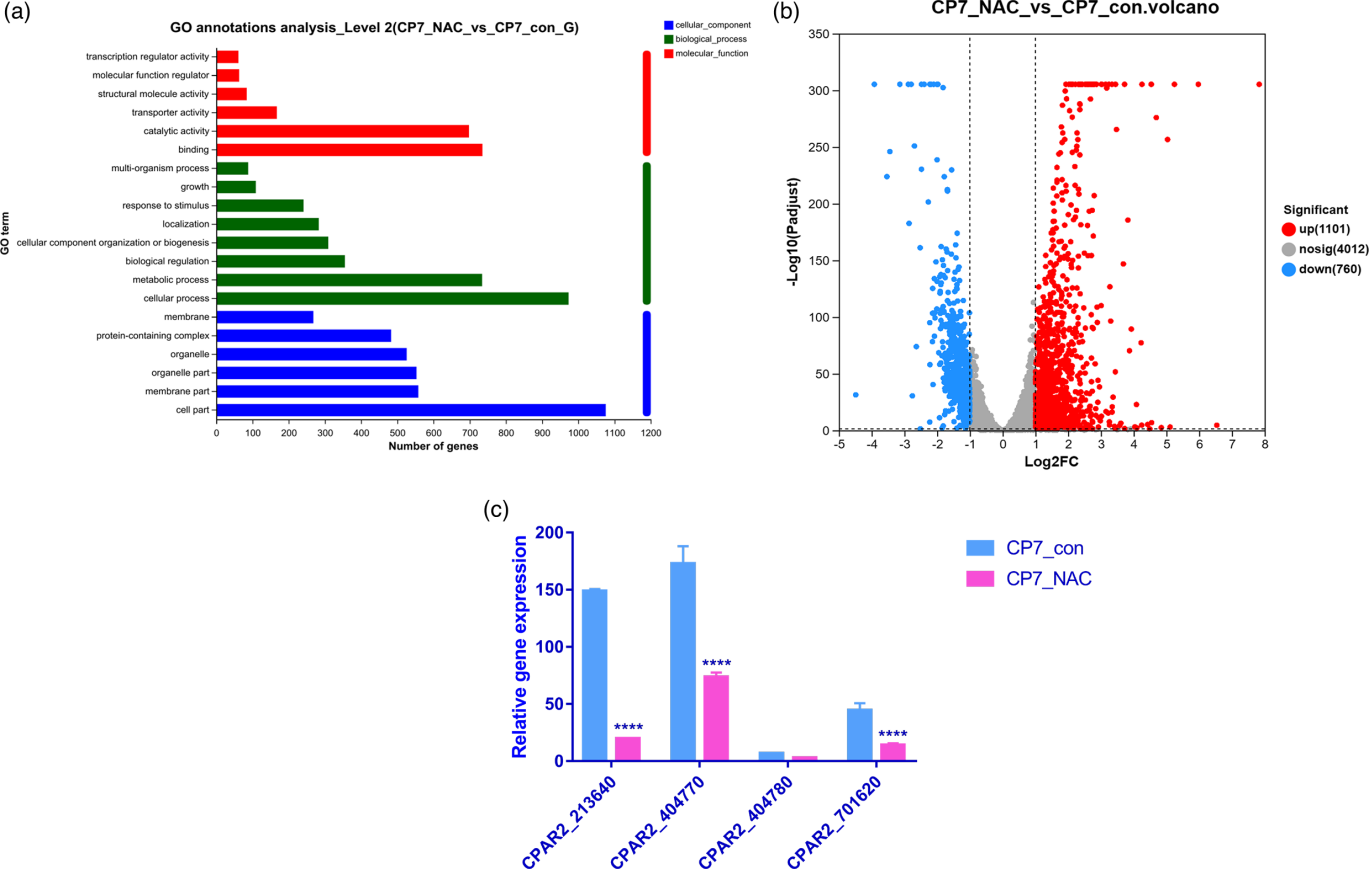
Transcriptome of *C. parapsilosis* clinical strains treated with NAC. (a) Gene Ontology annotation analysis of differentially expressed genes regulated by NAC. The significantly enriched pathways are shown. (b) Volcano map depicting the transcriptomics response to NAC. (c) Adhesion and biofilm forming-related genes are regulated by NAC. CPAR2_701620, CPAR2_213640, CPAR2_404770 and CPAR2_404780 are the gene names of factors EFG1, NDT80, ALS3 and ALS11 (gene id), respectively.

The transcriptome data after the intervention of NAC suggested that the metabolism of cell structure and biofilm forming factors was regulated (Fig. 5).

## Discussion

Our previous studies indicated a strong relationship between *C. parapsilosis* infections with an enhanced capacity to adhere to surfaces and form biofilms and critically ill patients who required prolonged use of CVCs. These studies suggest that biofilm formation may play an important role in CRBSIs caused by *C. parapsilosis* [16,20].

In recent years, the role of non-antibiotic preparations in antifungal biofilms has attracted increasing attention from clinical researchers. NAC is a precursor of intracellular cysteine and GSH that contains sulfhydryl groups, which provide protection by scavenging reactive oxygen species (ROS). There is also evidence that NAC improves the functional defects of mitochondria in rat hepatocytes by partially restoring the level of mitochondrial autophagy and inhibiting the formation of both cellular and mitochondrial ROS (mtROS), thereby preventing the onset of inflammation [21]. Due to this ability to act as a ROS quenchant, the effectiveness of NAC to inhibit biofilm formation and destroy the biofilm has been extensively studied [ 13,15, 22, 23].

Based on this potential of NAC to inhibit biofilm formation by *Candida*, six clinical *C. parapsilosis* strains with strong biofilm formation ability were screened in the early stages of the current study, followed by the construction of an *in vitro* biofilm model. The anti-biofilm effect of NAC was detected using the menadione method, crystal violet staining, flow cytometry and other experimental methods.

Our previous studies confirmed that *C. parapsilosis* clinical strains isolated from bloodstream infections had a strong ability to form biofilms [16]. The aim of the current study was to determine whether the anti-biofilm agent NAC had specific effects on the development of biofilms by *C. parapsilosis*. As anticipated, the study confirmed that NAC had an inhibitory effect on adhesion, biofilm formation and cell activity in the biofilm formed *in vitro* by *C. parapsilosis* (Figs1  2).

The key trigger interaction in the adhesion process is promoted by adhesins. Studies in *C. parapsilosis* have shown that five Als proteins are involved in adherence, with the ortholog CaAls7 having been described as a determinant for adhesion to host cells [24,25]. The process of biofilm development also involves such phenomena as the control of adhesion, morphology changes and ECM production. All these factors require an extensive and complex regulatory network [26]. Genes such as *BCR1*, *EFG1* and *NDT80* have been shown to have an important role in biofilm development in *C. parapsilosis*, with one recent report identifying *NDT80* as a repressor of *C. parapsilosis* virulence [26,27]. The current study therefore used qRT-PCR to detect the expression of adhesion-related genes (CpALS6 and CpALS7) and biofilm formation-related genes (CpEFG1 and CpBCR1) in *C. parapsilosis* after NAC intervention. The results showed the expression of the biofilm factor EFG1 was down-regulated (Fig. 4), indicating that NAC plays a role in inhibiting biofilm formation of *C. parapsilosis* and is therefore a potentially effective drug for the treatment of biofilm formation.

Transcriptomic and proteomic profiles have been widely used in the analysis of *C. parapsilosis* infections [28].Transcriptomics profiling during NAC exposure showed that the response of C. *parapsilosis* was quite extensive, mainly including the activation of biological processes such as regulation of biological, metabolic and cellular processes, cellular components and molecular function (Fig. 5a). As shown in Fig. 5b, our study showed a large transcriptional response with 1101 genes upregulated and 760 genes downregulated. A check of the adhesion- and biofilm-related genes such as EFG1 (CPAR2_701620), NDT80 (CPAR2_213640), ALS3 (CPAR2_404770) and ALS11 (CPAR2_404780) showed a trend of decreased expression for all these genes after incubation with NAC (Fig. 5c).

## Conclusions

The non-*C. albicans* species, *C. parapsilosis* exhibited a strong ability to adhere and form biofilms in patients with candidemia and catheter-related infections. The qRT-PCR results confirmed that the expression of *CpALS7*, *CpEFG1* and *CpBCR1* may play key roles in the adhesion and biofilm formation processes in *C. parapsilosis* CRBSIs.

NAC showed a strong inhibitory effect against both adhesion and biofilm formation in clinical *C. parapsilosis* strains isolated from CRBSIs, with this inhibitory effect being dose-dependent. NAC down-regulated the transcription levels of *CpEFG1* in * C. parapsilosis* clinical strains, and therefore may be a potential candidate for treatment of *C. parapsilosis* CRBSIs. Transcriptomics indicated that several genes involved in the biological, metabolic and cellular processes, cellular components and molecular functions were regulated. The pathways associated with this regulation should therefore be considered in future research studies.

## Abbreviations

CRBSI: catheter-related bloodstream infection
CRBSI: catheter-related bloodstream infection
CV: crystal violet staining
CVC: central venous catheter
dTTP: 2'-deoxythymidine 5'-triphosphate
dUTP: 2'-deoxyuridine 5'-triphosphate
ECM: extracellular matrices
FZ: fluconazole
GO: Gene Ontology
MALDI-TOF-MS: matrix-assisted laser desorption/ionization time-of-flight mass spectrometry
mtROS: mitochondrial ROS
NAC: *N*-acetylcysteine
qRT-PCR: quantitative reverse transcription
RHWU: Renmin Hospital of Wuhan University
ROS: reactive oxygen species
SDB: Sabouraud dextrose broth
SRA: Sequence Read Archive
XTT: 2,3-bis(2-methoxy-4-nitro-5-sulfophenyl)-5-toluidine-2H-tetrazolium
